# National Survey on Access, Use and Promotion of Rational Use of Medicines (PNAUM): evaluation of pharmaceutical services in the primary health care

**DOI:** 10.11606/S1518-8787.201705100supl2ed

**Published:** 2017-09-22

**Authors:** Marco Akerman, Osvaldo de Freitas

**Affiliations:** I Departamento de Política, Gestão e Saúde. Faculdade de Saúde Pública. Universidade de São Paulo. São Paulo, SP, Brasil; II Departamento de Ciências Farmacêuticas. Faculdade de Ciências Farmacêuticas. Universidade de São Paulo. Ribeirão Preto, SP, Brasil

The rational use of medicines is a goal that should not be attributed only to the meeting between health professional and user, because it goes beyond the limits of mere interaction between prescription and use that takes place in the doctor-patient relationship.

This alleged rationality in the use of medicines would require a difficult alignment between distinct rationalities of the various actors that interact in the field of pharmaceutical services: federal, state, and municipal governments; universities and technical courses; health professionals; public and private health services; pharmaceutical industry and providers of supplies; patients and media.

In this sense, the proposition “I think, therefore I am,” which would indicate the prominence of the Empire of reason, might fail, because “we think where we do not exist, or exist where we do not think”^23^.

However, this cannot move us away from the pursuit of a common vision that articulates the different actors of the field around a set of principles and agreements that can promote a quality use of medicines, namely: (1) strengthening the right to health care by universal health systems; (2) providing care with solutions in line with the needs of the population; (3) encouraging effective procedures; (4) promoting health and preventing diseases; and (5) setting in a transparent way the roles of the actors in the field.

Thus, we seek to reverse the logic that the medicine is only one “input” in the health care network, so that it can be seen as element of an interconnected chain that produces quality “outcomes”^8^.

To do so, we need: (1) easy access to essential medicines at costs that society can endure; (2) medicines that meet criteria of quality, safety, and efficacy; (3) quality use of medicines; (4) stimulus to industry segments that are viable and responsible^8^.

In this context, governments and universities gathered around the *Pesquisa Nacional sobre Acesso, Utilização e Promoção do Uso Racional de Medicamentos* (PNAUM – National Survey on Access, Use and Promotion of Rational Use of Medicines) to produce a comprehensive scenario of the situation of access and use of medicines in Brazil^12^.

*Revista de Saúde Pública* has published, in previous supplement^6^, results of the comprehensive household survey of PNAUM, which collected important information regarding several aspects of access and use of medicines: in childhood; among older people; for chronic diseases; free supply; for treatment of hypertension; and generic medicines. It also investigated the catastrophic health expenses, self-medication, factors affecting the adherence to treatment, and prescription of contraceptives.

This Supplement deals with the evaluation of pharmaceutical services in the primary health care – indicating advances and challenges –; the methods used; the concepts of what is meant by pharmaceutical services; clinical pharmaceutical activities carried out; the way how pharmaceutical services are being institutionalized in the cities; and how access to medicines occurs. Still, this supplement explores how the organization of the workforce, of the selection, availability, and dispensation of essential drugs in primary health care, and of the funding and management of pharmaceutical services takes place. Also, it examines patient-related aspects – access, use, rational use, polypharmacy, satisfaction, and quality of life.

Costa et al.^13^ synthesize the main findings based on a critical narrative of the elements of pharmaceutical policies in Brazil. Despite the advances, which reflect the commitment of the group of actors involved, the results of the survey indicate challenges such as equitable access to medicines, the structuring of pharmaceutical services, the improvement of logistics and management, and the implementation of actions directed to pharmaceutical care in the health units. Álvares et al.^1^ describe the planning and methodology used in PNAUM in a clear, objective, and detailed way, from the sample calculation and actors involved to the implementation in the field.

Although there are initiatives in the management of the Brazilian Unified Health System (SUS) to create some degree of standardization of procedures, Costa et al.^10^ show a great diversity of understandings of pharmaceutical services by the actors related to it. However, it was identified, in the process of its reorientation, an initiative that reflects a gradual shift from the technical paradigm, focusing on medicine logistics, to a patient-oriented approach by the health services.

However, Araújo et al.^3^ report that activities of clinical nature performed by pharmacists are still incipient in Brazil. This can be attributed to the lack of appropriate place and to the small participation in educational activities of health promotion, suggesting little integration of pharmacists with the health team and of pharmaceutical services with other health actions.

Souza et al.^25^ characterize the current stage of institutionalization of pharmaceutical services in Brazilian cities, which is heterogeneous and partial, showing regional inequalities. Therefore, Barros et al.^5^ show that access to medicines was higher in places with the following dimensions: management tools; participation and social control; funding and structure.

And how are medicines selected for the health care network? Karnikowski et al.^17^ presented the first national survey that characterizes the process of selection of medicines within primary health care. All actors interviewed claimed to have a list of essential medicines, but which only partially meets health demands. Thus, Nascimento et al.^21^ show that, despite all efforts, it is still a challenge to ensure access to essential medicines in the context of primary health care of SUS, and they offer subsidies for the improvement of pharmaceutical services in the public network.

In addition, Leite et al.^18^ observed large differences in the models of dispensing organization. The centralization of medicine dispensation in isolated pharmacies of the health services is associated with better structural and professional conditions, as in the dispensing units of the South, Southeast, and Midwest. However, the development of dispensation as health service not yet prevails in any pharmacy or region of the Country.

Regardless of the features in the selection and dispensation, Costa et al.^11^ show that the health status of medicines in the Brazilian primary health care is worrying, in view of the breach of the specific health legislation for dispensing facilities. The authors also described a broad set of essential requirements for the conservation of medicines and a gap between the efforts to promote access and the organization and qualification of pharmaceutical services. In this sense, Leite et al.^19^ suggest that the environment of pharmaceutical services needs to be restructured so as to enable the humanization of care and better working conditions for professionals.

Without adequate financial resources, there is no way to improve the selection and dispensing aspects and the quality of conservation and inventory of medicines. Faleiros et al.^14^ discuss the funding of pharmaceutical services and address factors related to the financing of the Basic Component of Pharmaceutical Services within the municipal management of SUS, in 600 cities distributed in the five regions of the Country. Results show serious shortcomings in the management, insufficiency of resources intended for the Basic Component of Pharmaceutical Services, and exhaustion of the financing model. The management is also addressed by Gerlack et al.^15^, by identifying that, unfortunately, the guidelines dictated by both the legal and political framework of pharmaceutical services are not met.

And how do health professionals act before this profile established so far? Carvalho et al.^7^ observe important shortcomings in the workforce composition in dispensing units, which may impair the quality of the use of medicines and its results on the health of the population.

Patients, which are the main reason of health services, are treated in many ways.

Interviews were carried out with 8,803 patients distributed in different parts of the Country^16^. Most were women (75.8%). Half of patients were classified as class C and 24.8% received the *Bolsa Familia* benefit. Only 9.8% had health insurance, with higher proportion in the South region and lower in the North and Midwest. Alcohol consumption and smoking were more present among men. Costa and Silveira^9^ deepened the analysis and noted that 76.2% of patients reported having used medicines (2.3 on average) in the stipulated period. In general, these patients were people with low education level and with comorbidities, especially older people (65 years or more). This group also reported more difficulty in the use of medicines, making them more vulnerable.

Nascimento et al.^22^ identified that polypharmacy (use of five or more medicines) is a reality for the population attended within the primary health care of SUS, and it may be related to the excessive or inappropriate use of medicines.

From the patients’ perspective, Álvares et al.^2^ compare the results of the evaluation of access to medicines in primary health care of SUS with those of developed countries. However, access remains a challenge, because it is still heavily impaired by the low availability of essential medicines in public health units.

Soeiro et al.^24^ showed the satisfaction of patients with pharmaceutical services: the overall percentage was 58.4% (95%CI 54.4–62.3), with emphasis on the dimension of interpersonal aspects, with 90.5% (95%CI 88.9–91.8).

In addition to the use of medicines, Ascef et al.^4^ analyzed the quality of life of patients of primary health care, discussed the predominant factors, and suggested that this indicator may guide a comprehensive care and health promotion actions.

The article by Lima et al.^20^ ends the supplement and shows that the results of the evaluation of the indicators regarding rational use of medicines and their associated factors in basic health units vary significantly between the regions of Brazil.
